# Targeting of neuroinflammation by glibenclamide in Covid-19: old weapon from arsenal

**DOI:** 10.1007/s10787-022-01087-8

**Published:** 2022-11-23

**Authors:** Gaber El-Saber Batiha, Hayder M. Al-kuraishy, Ali I. Al-Gareeb, Mubarak Alruwaili, Raed AlRuwaili, Sarah M. Albogami, Mohammed Alorabi, Hebatallah M. Saad, Jesus Simal-Gandara

**Affiliations:** 1 Department of Pharmacology and Therapeutics, Faculty of Veterinary Medicine, Damanhour University, Damanhour, 22511 AlBeheira Egypt; 2 Professor in department of clinical pharmacology and medicine, College of Medicine, Mustansiriyah University, Baghdad, Iraq; 3 Department of Internal Medicine, College of Medicine, Jouf University, Sakaka, Saudi Arabia; 4 Department of Biotechnology, College of Science, Taif University, P.O.Box 11099, Taif, 21944 Saudi Arabia; 5 Department of Pathology, Faculty of Veterinary Medicine, Matrouh University, Marsa Matruh, 51744 Egypt; 6 Nutrition and Bromatology Group, Department of Analytical Chemistry and Food Science, Faculty of Science, Universidade de Vigo, E-32004 Ourense, Spain

**Keywords:** Covid-19, Neuroinflammation, Glibenclamide

## Abstract

In coronavirus disease 2019 (Covid-19) era, neuroinflammation may develop due to neuronal tropism of severe acute respiratory syndrome coronavirus type 2 (SARS-CoV-2) and/or associated immune activation, cytokine storm, and psychological stress. SARS-CoV-2 infection and linked cytokine storm may cause blood–brain barrier (BBB) injury through which activated immune cells and SARS-CoV-2 can pass into the brain causing activation of glial cells with subsequent neuroinflammation. Different therapeutic regimens were suggested to alleviate Covid-19-induced neuroinflammation. Since glibenclamide has anti-inflammatory and neuroprotective effects, it could be effective in mitigation of SARS-CoV-2 infection-induced neuroinflammation. Glibenclamide is a second-generation drug from the sulfonylurea family, which acts by inhibiting the adenosine triphosphate (ATP)-sensitive K channel in the regulatory subunit of type 1 sulfonylurea receptor (SUR-1) in pancreatic β cells. Glibenclamide reduces neuroinflammation and associated BBB injury by inhibiting the nod-like receptor pyrin 3 (NLRP3) inflammasome, oxidative stress, and microglial activation. Therefore, glibenclamide through inhibition of NLRP3 inflammasome, microglial activation, and oxidative stress may attenuate SARS-CoV-2-mediated neuroinflammation.

## Introduction

Coronavirus disease 2019 (Covid-19) represents a current pandemic disease caused by a novel severe acute respiratory syndrome coronavirus type 2 (SARS-CoV-2) leading to formidable global effects (Al-kuraishy et al. 2022c; Al-Thomali et al. 2022). Covid-19 is regarded as a primary respiratory disease leading to a mild respiratory infection. However, in severe cases, it causes acute lung injury (ALI) and the development of acute respiratory distress syndrome (ARDS) (Al-kuraishy et al. 2022a; Al-kuraishy et al. 2022e). Besides, Covid-19 may cause extra-pulmonary complications including acute kidney injury, stroke, hepatic injury, testicular injury and neuroinflammation due to the propagation of hyper-inflammation and cytokine storm (Al-kuraishy et al. 2022b).

Furthermore, in Covid-19, neuroinflammation may develop due to neuronal tropism of SARS-CoV-2 and/or associated immune activation, cytokine storm, and psychological stress (Kempuraj et al. 2020; Ojo et al. 2021; Alorabi et al. 2022). Exaggeration of peripheral immune response and hyper-inflammation in SARS-CoV-2 infection can exacerbate and causes neuroinflammation by activating mast cells (Kempuraj et al. 2020; Koneru et al. 2021). SARS-CoV-2 infection and linked cytokine storm may cause blood–brain barrier (BBB) injury through which activated immune cells and SARS-CoV-2 can pass into the brain causing activation of glial cells with subsequent neuroinflammation (Pacheco-Herrero et al. 2021). In the clinical setting, it has been reported that the latency of about one week gap between the onset of severe Covid-19 and onset of neuroinflammation due to SARS-CoV-2 and alteration in the function of BBB (Pacheco-Herrero et al. 2021). SARS-CoV-2-induced neuroinflammation is associated with increased biomarkers of neuronal and BBB injuries such as neurofilament light chain (NfL) and glial fibrillary acidic protein (GFAP) in Covid-19 patients (Kanberg et al. 2020). A cross-sectional study included 47 Covid-19 patients with mild (*n* = 20), moderate (*n* = 9) and severe (*n* = 18) showed that patients with severe Covid-19 had higher levels of GFAP and NfL compared to the mild and moderate ones. The early peak of GFAP was reduced on follow-up while NfL remained high during the follow-up (Kanberg et al. 2020). This finding suggests that SARS-CoV-2-induced neuroinflammation is linked with early astrocyte activation and delayed axonal injury in Covid-19. SARS-CoV-2-induced neuroinflammation is more common in the elderly due to low-grade inflammatory changes which might explain the greater risk of Covid-19 in the elderly age group (Bossù et al. 2020).

Baig and other studies revealed that SARS-CoV-2 can be transported through general circulation and enters the brain via cerebral microcirculation where it binds angiotensin-converting enzyme 2 (ACE2) in the neurovascular unit leading to the induction of neuroinflammation (Baig et al. 2020; Abubakar et al. 2021; Babalghith et al. 2022). It has been reported that 36.4% of Covid-19 patients presented with neurological manifestations including dizziness, headache, impaired consciousness, and cerebrovascular events (Mao et al. 2020). Similarly, a prospective study showed that 13.5% of Covid-19 patients had neurological symptoms associated with poor clinical outcomes and high mortality (Frontera et al. 2021; Mathew et al. 2021).

Different therapeutic regimens were suggested to alleviate Covid-19-induced neuroinflammation. Ong and colleagues suggested that antimalarial drugs could be effective in the management of SARS-CoV-2 infection-induced neuroinflammation by inhibiting phospholipase A2 (PLA2) (Ong et al. 2021). Besides, selective serotonin reuptake inhibitor fluvoxamine which has an agonist effect on the sigma-1 receptor was confirmed in a randomized clinical trial to be effective in reducing Covid-19-induced neuroinflammation and clinical deterioration (Lenze et al. 2020; Al-kuraishy et al. 2021a). In addition, statins were proposed recently to be effective against Covid-19-induced neuroinflammation and acute brain injury by their anti-inflammatory effects (Hussien et al. 2021; Alsubaie et al. 2022). Of note, glibenclamide has anti-inflammatory and neuroprotective effects (Hussien et al. 2018) therefore we hypothesized that glibenclamide could be an effective agent in the mitigation of SARS-CoV-2 infection-induced neuroinflammation.

### Glibenclamide and neuroinflammation

**Fig. 1:**
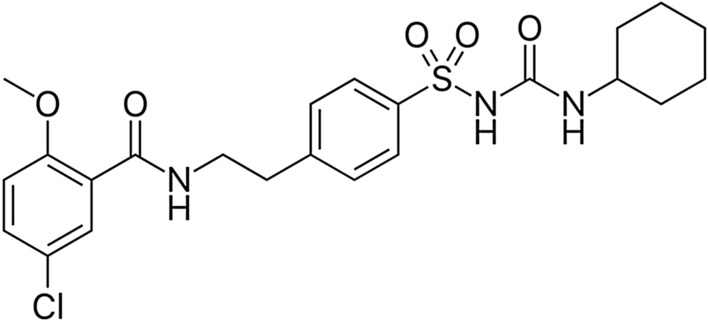
Chemical structure of glibenclamide

Glibenclamide is a second-generation drug from the sulfonylurea family (Fig. 1), which acts by inhibiting the adenosine triphosphate (ATP)-sensitive K channel in the regulatory subunit of type 1 sulfonylurea receptor (SUR-1) in pancreatic β cells. This effect induces membrane depolarization with increasing intracellular Ca^+2^ within pancreatic β cells and subsequent insulin release (Najdi et al. 2019). Glibenclamide is mainly used in the management of type 2 diabetes mellitus (T2DM); however, it is not the first-line therapy in T2DM (Najdi et al. 2019; Batiha et al. 2022).

Glibenclamide is regarded as an old drug; it was discovered in 1969 and permitted in medical use in 1984 (Katsilambros 2006). There are three isoforms of SUR, SUR-1 in the pancreatic β cells, SUR-2A, and SUR-1B in the heart and adipose tissue, respectively, though the brain expresses all types of SUR isoforms (Katsilambros 2006). It has been reported that neuroinflammation was associated with over-expression of SUR-1 and tumor necrosis factor-alpha (TNF-α) (Simard et al. 2012; Batiha et al. 2021). The use of glibenclamide can reduce neuroinflammation in experimental rats through the inhibition of SUR-1 (Tosun et al. 2013). Hussien et al (2018) proposed the neuroprotective effects of glibenclamide in reducing ischemic stroke and brain edema through induction of neurogenesis and possible anti-inflammatory effects. Similarly, glibenclamide was suggested to be a potent systemic anti-inflammatory agent against respiratory, cardiac, digestive, and neurological inflammation by its anti-inflammatory effects with reduced release of pro-inflammatory cytokines (Zhang et al. 2017). A recent experimental study observed that glibenclamide mitigated hippocampal inflammation and cognitive impairment in T2DM rats (Esmaeili et al. 2020). Zhang and colleagues revealed that glibenclamide had a protective effect against inflammation-mediated neuronal injury (Zhang et al. 2017). Similarly, glibenclamide promotes neurological recovery and neuroinflammation following intracerebral hemorrhage in rats (Jiang et al. 2021). Notably, oral treatment of glibenclamide can mitigate functional outcomes in patients with moderate to severe traumatic brain injuries (Khalili et al. 2017).

These verdicts suggest that glibenclamide is effective against neuroinflammation by its anti-inflammatory effects. Depending on these observations and suggestions, glibenclamide may be effective against viral infections including SARS-CoV-2.

### Glibenclamide and Covid-19-induced neuroinflammation

In severe SARS-CoV-2 infection, different inflammatory signaling pathways are activated with the subsequent release of pro-inflammatory cytokines including TNF-α, interleukins (IL-1β, IL-6) and chemokines (Mostafa-Hedeab et al. 2022; Al-Kuraishy et al. 2022d).

Of note, in Covid-19-induced neuroinflammation, nod-like receptor pyrin 3 (NLRP3) inflammasome is activated by SARS-CoV-2 and/or activated microglial cells causing progressive neuroinflammation and brain injury (Cama et al. 2021; Ezeonuegbu et al. 2021). Thus, suppression of NLRP3 inflammasome could be an effective strategy against the development of SARS-CoV-2-induced neuroinflammation (Cama et al. 2021). Likewise, activation of the NLRP3 inflammasome by SARS-CoV-2 is linked with the development of BBB injury (Zhao et al. 2021). Severe disruption of BBB in Covid-19 leads to critical neuroinflammation and central nervous system (CNS) complications (Welcome and Mastorakis 2021). Targeting of NLRP3 inflammasome in SARS-CoV-2 by specific inhibitors can mitigate the neuroinflammation and associated BBB injury in Covid-19 patients (Freeman and Swartz 2020). As well, natural products such as Oridonin, Parthenolide and vinyl sulfone-related compounds have potential inhibitory effects on the activation of NLRP3 inflammasome (Shah 2020).

Of interest, glibenclamide reduces neuroinflammation and associated BBB injury by inhibiting NLRP3 inflammasome in mice with experimental intracerebral hemorrhage (Xu et al. 2019). Yang et al (2019) found that glibenclamide had a neuroprotective effect through inhibition of the NLRP3 inflammasome signaling pathway. In addition, glibenclamide can decrease microglial activation-induced neuroinflammation through the suppression release of pro-inflammatory cytokines IL-1β, IL-6, and TNF-α (Esmaeili et al. 2020; Mahran et al. 2021).

Glial cells including microglia and astrocytes respond to the brain insults-induced neuroinflammation and could be a potential target of SARS-CoV-2 due to higher expression of ACE2 (McMahon et al. 2021). As well, glial cells are the major source of inflammatory cytokines in the CNS (Vargas et al. 2020). In general, glial cells are involved in viral clearance through the recruitment of immune cells and the activation release of antiviral cytokines (Amaral et al. 2021). However, over-activation of microglial cells by the persistence of viral infection or through activation of astrocytes induces the release of pro-inflammatory cytokines with the development of cytokine storm which causes synaptic loss and BBB injury (Mangale et al. 2020; Opara et al. 2021). Therefore, microglial cells might be responsible for direct neuronal injury or T cell/astrocytes-induced neurotoxicity and cytokine storm. Glibenclamide had a neuroprotective role with the reduction of cerebral edema and the release of inflammatory cytokines by inhibiting glial and microglial cells in rats (Kajimoto et al. 2022).

These findings suggest that glibenclamide may reduce Covid-19-induced neuroinflammation through its anti-inflammatory effects which are mediated by suppressing the activation of NLRP3 inflammasome and the release of pro-inflammatory cytokines.

Furthermore, the induction of oxidative stress during SARS-CoV-2 infection may cause neuroinflammation and other CNS complications through the induction of nuclear factor kappa B (NF-κB) (Karnik et al. 2021). Indeed, glibenclamide inhibits neuronal ischemic-reperfusion injury in rat hippocampus through suppression the development of oxidative stress (Abdallah et al. 2011; Yaqoob et al. 2021). Different experimental studies demonstrated that glibenclamide can attenuate acute brain injury by  reducing the generation of reactive oxygen species (ROS) and inducing the expression of antioxidant enzymes (Abdallah et al. 2011; Erejuwa et al. 2010). However, the effect of glibenclamide on the redox potential cellular changes in relation to neuroinflammation is not well-defined. Thus, the glibenclamide effect on SARS-CoV-2 infection needs further studies.

Of interest, glibenclamide was not evaluated in Covid-19 patients as most critical diabetic patients switched to insulin therapy for strict glucose control (Rodrigues Ferreira et al. 2021; Al-kuraishy et al. 2021b). In addition, the use of glibenclamide in diabetic Covid-19 patients may increase the risk of hypoglycemia (Nakhleh and Shehadeh 2020). Of note, the medical use of glibenclamide is associated with the risk of hypoglycemia in diabetic and non-diabetic patients (Soydan et al. 2013). However, the use of a non-hypoglycemic dose of glibenclamide 0.4 mg/kg still has an anti-inflammatory effect (Berdugo et al. 2021). Therefore, the use of a lower effective dose of glibenclamide could be a promising therapeutic strategy in the management of SARS-CoV-2-induced neuroinflammation even in non-diabetic patients. Remarkably, glibenclamide is effective against ALI by inhibiting the release of pro-inflammatory cytokines and the generation of ROS (Nakhleh and Shehadeh 2020; Moubarak et al. 2021). In this state, glibenclamide can decrease the risk for the development of hypoxemia which is associated with the propagation of neuroinflammation and acute brain injury (Amruta et al. 2021).

Moreover, downregulation of ACE2 by SARS-CoV-2 may induce dysregulation of the renin-angiotensin system (RAS) with increasing vasoconstrictor angiotensin II (AngII) and reduction of vasodilator Ang1-7 (Al-Kuraishy et al. 2021c; Alkazmi et al. 2022). Dysregulated RAS in Covid-19 increases the risk for the development of cytokine storm and other critical complications including ALI (Al-Kuraishy et al. 2021d). As well, hyperglycemia in T2DM may trigger dysregulation of RAS causing propagation of diabetic complications (Nakhleh and Shehadeh 2020). Interestingly, insulin therapy induces the expression of a protective anti-inflammatory ACE2, which may reduce Covid-19 complications in T2DM patients (Nakhleh and Shehadeh 2020). Since glibenclamide stimulates insulin release from pancreatic β-cells (Hussien et al. 2018), a lower dose of glibenclamide may decrease Covid-19 complications through the insulin-mediated pathway.

**Fig. 2:**
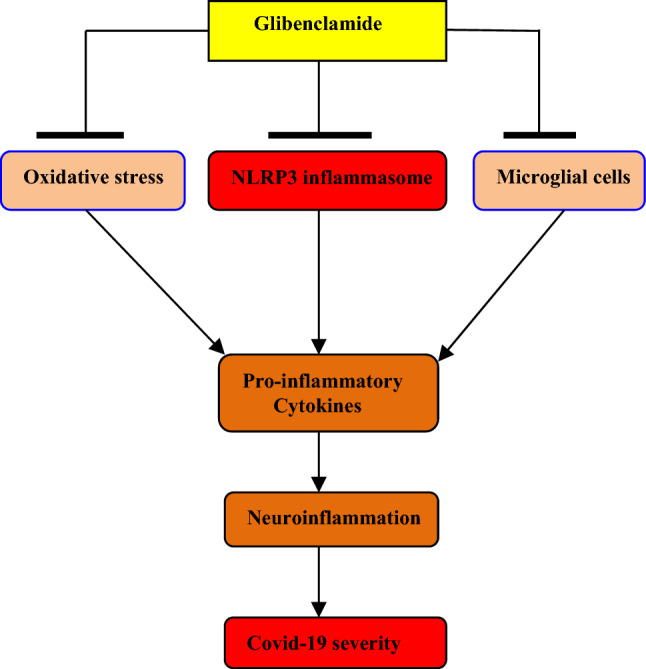
The possible role of glibenclamide against neuroinflammation in Covid-19

Therefore, glibenclamide through inhibition of NLRP3 inflammasome, microglial activation, and oxidative stress may attenuate SARS-CoV-2-mediated neuroinflammation (Fig. 2).

These findings and our hypotheses provoke future preclinical and clinical studies to confirm the potential role of glibenclamide in Covid-19 and associated neuroinflammation. However, the present hypotheses had many limitations including a paucity of clinical studies regarding the use of glibenclamide in patients with neuroinflammation. Besides, glibenclamide is little to be used in T2DM patients with Covid-19 as most of them switched to insulin therapy mainly in the severe state. Therefore, experimental, preclinical and clinical studies are warranted in this regard to confirm the lower non-hypoglycemic dose of glibenclamide in Covid-19 even in non-diabetic patients.

## Conclusions

Glibenclamide is an antidiabetic drug used in the management of T2DM by inhibiting ATP-sensitive K channel SUR-1 in pancreatic β cells. Glibenclamide reduces neuroinflammation and associated BBB injury by inhibiting NLRP3 inflammasome, oxidative stress, and microglial activation. Therefore, glibenclamide may attenuate SARS-CoV-2-mediated neuroinflammation.

## Data Availability

All data are included in this manuscript.
